# Vertical transmission of hepatitis E virus in pregnant rhesus macaques

**DOI:** 10.1038/s41598-020-74461-7

**Published:** 2020-10-15

**Authors:** Wenhai Yu, Xianhui Hao, Yi Li, Chenchen Yang, Yunlong Li, Zhanlong He, Fen Huang

**Affiliations:** 1 Institute of Medical Biology, Chinese Academy of Medical Sciences and Peking Union Medical College, Kunming, People’s Republic of China; 2 Medical School, Kunming University of Science and Technology, Kunming, People’s Republic of China

**Keywords:** Immunology, Microbiology, Zoology, Pathogenesis

## Abstract

Hepatitis E virus (HEV) is the major pathogen of viral hepatitis. HEV causes high mortality in pregnant women. Its infection during pregnancy usually leads to fulminant hepatic failure, spontaneous abortions, premature delivery, or stillbirth. Vertical transmission of HEV has been reported, but the pathogenesis during pregnancy remains largely elusive. Pregnant rhesus macaques were infected with HEV to explore the pathogenesis of genotype 4 HEV infection during pregnancy. Active HEV infections were established with shedding viruses in the feces and blood, and elevated liver enzymes. Notably, higher viral titers and longer durations of HEV infection were found in HEV-infected pregnant rhesus macaques than in non-pregnant macaques. Premature delivery and fetal death occurred in one of the HEV-infected pregnant rhesus macaques. HEV RNA was detected in the liver, spleen, kidneys, and intestines of the dead fetus. This result strongly indicated vertical HEV transmission from mother to fetus. Maternal-transferred antibodies were observed in one of the babies with poor protection. The expressions of interferon-stimulated genes (ISGs) related to HEV infection were completely different between pregnant and non-pregnant rhesus macaques. During pregnancy, impaired innate immune responses, reduced progesterone levels, and shifts in immune states may aggravate HEV infection and result in adverse pregnancy outcomes.

## Introduction

Hepatitis E virus (HEV) infection is a serious public health issue that causes over 20 million infections and 7000 deaths annually^1^. In general populations, most HEV infections are asymptomatic and self-limited. However, HEV infection results in high mortality (> 20%) in pregnant women, especially during the third trimester of pregnancy^2,3^. The risk of HEV infection is substantially higher in pregnant women than in non-pregnant women^1,4^. Adverse pregnancy outcomes, such as fulminant hepatic failure (FHF), eclampsia, hemorrhage, abortion, and stillbirths due to HEV infection have been reported worldwide^2,5^. More importantly, about 46.09% of fetuses are infected by his/her mother^6^. However, the vertical transmission of HEV in pregnant women remains largely elusive.

HEV has eight recognized genotypes (gt) within the species Orthohepevirus A: gt1–gt8. Gt1 and gt2 HEV only infect humans and are prevalent in developing countries^1^. Gt3, gt4, and gt7 HEV are zoonotically transmitted from animals to humans; these genotypes are predominant in developed countries^7,8^. Previous studies have reported that gt1 or gt2 HEV during pregnancy causes acute liver failure and maternal death^9^. In China, adverse pregnancy outcomes, including preterm births, premature rupturing of membranes, and abortion, have been reported in genotype 4 HEV-infected pregnant women^10,11^. However, the pathogenesis of gt4 HEV in pregnant women is unclear.

Pregnancy is a complex physiological process. Successful pregnancy depends on the establishment and maintenance of an adequate maternal–fetal interface and maternal immune tolerance of semiallogeneic fetuses^12^. The immune system is suppressed to protect the fetus from immunological recognition and rejection. The immunosuppressed state during pregnancy possibly increases the risk of pathogen infection. Innate immune responses play a crucial role in defending against viral infections^13,14^. Virus infection induces innate immune responses in cells mainly through pathogen recognition receptors (PRRs), including retinoic acid-inducible gene I (RIG-I)-like receptors and Toll-like receptors^15^. The trigger of PRRs by virus invasion activates downstream signal cascades and various signaling molecules to express type I interferons (IFNs)^16,17^. IFN induces the expression of numerous IFN-stimulated genes (ISGs) via the JAK-STAT signaling pathway^18^; ISGs play an important role in antiviral immune responses^19^. However, the mechanism by which these ISGs change during pregnancy when infected by HEV is unknown.

An animal model is essential in exploring the pathogenesis of HEV infection during pregnancy. Rhesus macaque is the perfect animal model for both acute and chronic HEV pathogenesis studies^20,21^. In the present study, pregnant rhesus macaques were used to evaluate the vertical transmission of HEV.

## Results

### Establishment and characterization of HEV gt4 infection in pregnant rhesus macaques

**Figure 1:**
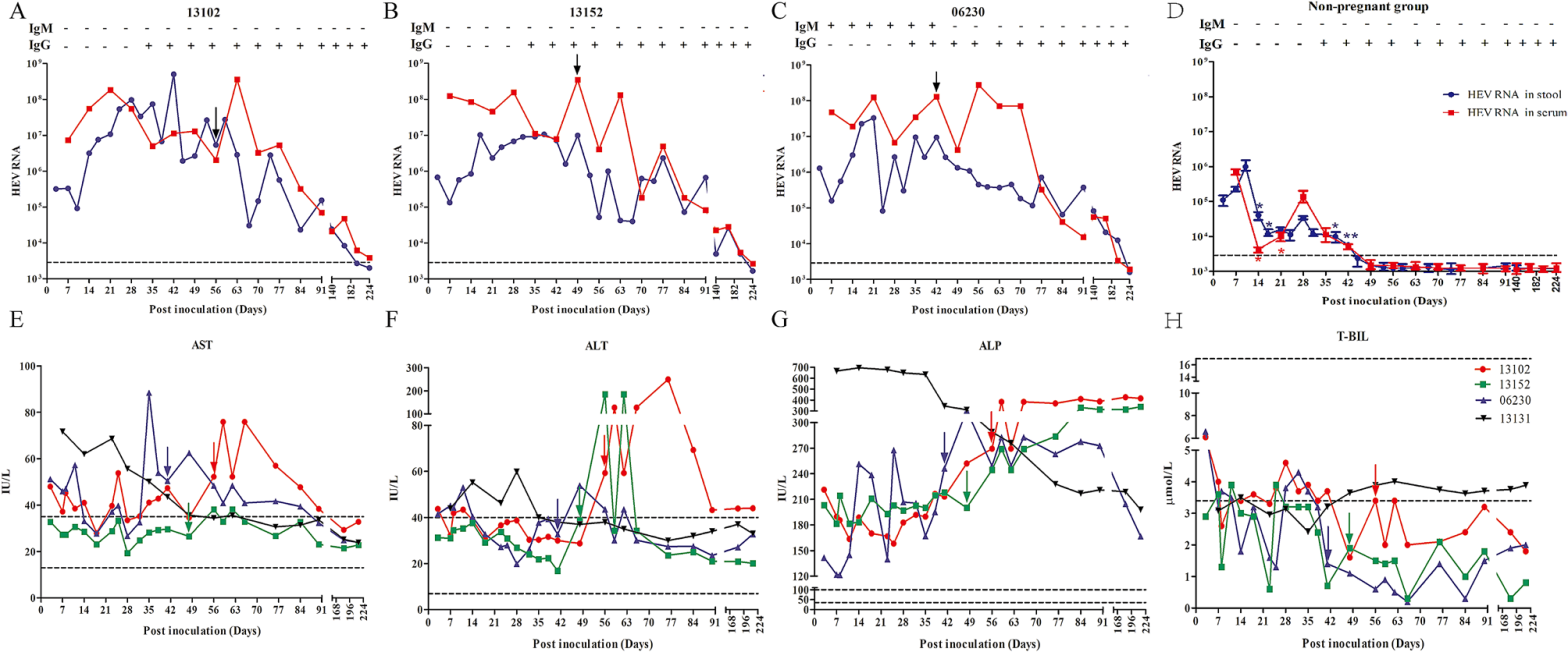
Profile of pregnant rhesus macaques infected with HEV. The copy number of HEV in the serum and stool in pregnant rhesus macaques was quantified by qRT-PCR, and anti-HEV IgG and IgM antibodies were determined by ELISA (**A**–**C**) or non-pregnant rhesus macaques (Mean ± SD of three non-pregnant HEV-infected rhesus macaques) (**D**). Asterisk (*) shown there is a significant difference of viral titers between pregnant rhesus macaques and non-pregnant rhesus macaques in indicated time points. *P < 0.05; **P < 0.01. Arrow indicated the time of delivery. The level/activity of liver enzymes AST (**E**), ALT (**F**), ALP (**G**), and T-BiL (**H**) was determined at indicated time points.

Rhesus macaque is the best animal model for HEV infection study. To explore the effects of gt4 HEV infection during pregnancy, we infected rhesus macaques in the third trimester of pregnancy with gt4 HEV (KM01 strain). Its infectivity was established in rhesus macaques and BALB/c mice in our previous studies^20,22^. Shedding of viruses in blood/stool samples and seroconversion are the classic diagnoses of HEV infection because asymptomatic patients are usually observed in clinical settings. Interestingly, HEV RNA was detectable in the feces at 4–7 days post-inoculation (dpi) in both HEV-infected pregnant and non-pregnant rhesus macaques. HEV RNA was negative at 49 dpi in non-pregnant rhesus macaques in both stool and serum samples (non-pregnant group, Fig. 1D), but persistently positive at over 196 dpi (more than 6 months) in pregnant rhesus macaques (196 dpi for 13102# and 224 dpi for both 13152# and 06230#) (Fig. 1A–C). Meanwhile, viremia was observed in all HEV-infected macaques. Viremia in acute hepatitis E generally persists for less than a month following symptom onset in otherwise healthy individuals. However, viruses in the serum samples of pregnant rhesus macaques lasted for 196 days, which was longer than that in non-pregnant macaques (42 days). Remarkably, viral titer in the serum samples of pregnant rhesus macaques was significantly higher than that in non-pregnant macaques at 14 dpi (425.79-fold, *P* = 0.04). Similarly, viral titer in the stool samples of pregnant rhesus macaques was 212.93-fold higher than that in non-pregnant macaques at 42 dpi (*P* = 0.01, Fig. 1D). These results were consistent with clinical observations that HEV-infected pregnant women usually bear a higher viral load than non-pregnant women^2^. Results strongly suggested that HEV infection successfully established in pregnant rhesus macaques. HEV replication is more violent in pregnant women than in non-pregnant women with a longer duration and a higher viral load.

HEV infection can activate humoral responses to product HEV-specific IgM and IgG antibodies. In the present study, anti-HEV IgG antibody was positive from 35 to 224 dpi (end of the experiment, Fig. 1A–D), which lasted for more than 7 months. However, only one of the infected macaques (06230#) was positive to anti-HEV IgM antibody from 7 to 42 dpi (Fig. 1C). In addition, elevated levels/activities of liver enzymes, including AST, ALT, ALP, T-BIL, D-BIL, I-BIL, GGT, TP, ALB, and GLB, were observed during HEV infection (Fig. 1E–H, Supplementary Fig. 1). Notably, relatively higher AST, ALT, ALP, T-BIL, and GGT levels were observed in non-pregnant rhesus macaques than in pregnant rhesus macaques during HEV infection.

To investigate the profiles of hematological parameters during gt4 HEV infection, we determined 24 hematological parameters in HEV-infected pregnant and non-pregnant rhesus macaques. Interestingly, the hematological parameters between HEV-infected pregnant and non-pregnant rhesus macaques were quite different. The levels of WBC, RBC, HGB, BCT, MPV, MCV, MCH, MCHC, LYMPH%, EO%, BASO%, PDW, and P-LCR in non-pregnant rhesus macaques were higher than those in HEV-infected pregnant macaques. By contrast, the levels of PLT, PCT, NEUT%, MONO%, RDW-SD, and RDW-CV in HEV-infected non-pregnant rhesus macaques were lower than those in pregnant rhesus macaques (Supplementary Fig. 2).

### Vertical HEV transmission from mother to fetus

Vertical transmission is one of the main transmission routes for HEV that causes premature births and prenatal mortality^9,10^. Mother-to-child transmission was observed in 46.09% (59/128) of HEV IgM-positive mothers^6^. Placenta is an extra-hepatic site for HEV replication, which was identified using a fetal placenta organ culture system^23^. However, the mechanism of vertical HEV transmission remains largely unknown.

**Figure 2:**
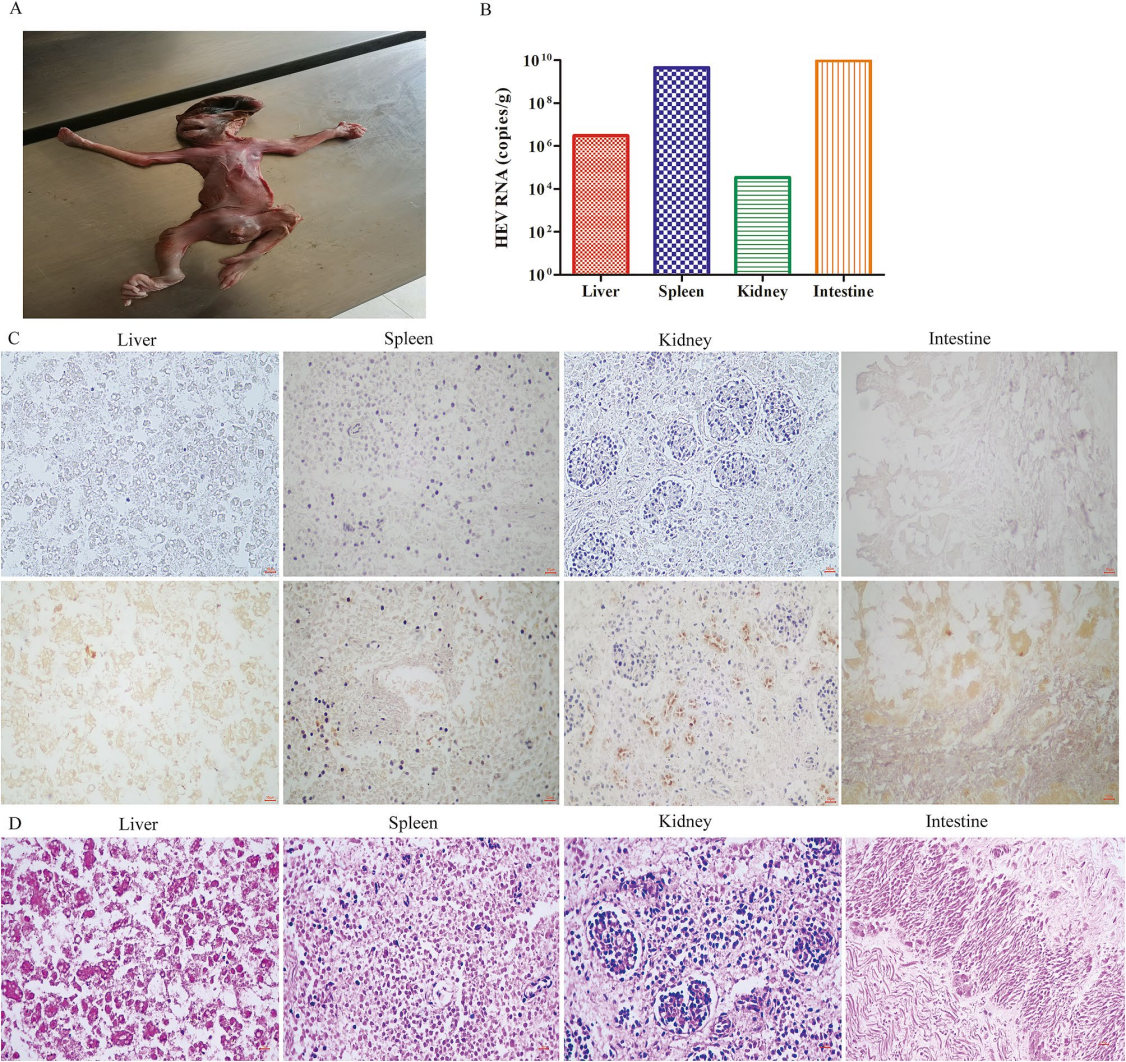
Vertical transmission of HEV from mother to fetus. The premature dead fetus born from HEV infected pregnant rhesus (06230#) (**A**). The viral titer of liver, spleen, kidney, and intestine of the dead fetus were quantified by qRT-PCR (**B**); HEV antigens were detected in the liver, spleen, kidney and intestine of the dead fetus by IHC inoculated with PBS (up panel) or HEV ORF2 specific antibody (down panel) (**C**). Histopathological analysis of liver, spleen, kidney, and intestine from the dead fetus (**D**).

In the present study, pregnant rhesus macaques were infected with HEV to determine whether genotype 4 HEV can be vertically transmitted from mother to fetus. Notably, one premature infant died immediately after delivery. Its mother (06230#) was infected with HEV 42 days before delivery. Thus, tissues were immediately collected from the dead fetus to evaluate the possibility of vertical HEV transmission. HEV RNA was detected in the liver, spleen, kidneys, and intestines of the premature fetus via qRT-PCR (Fig. 2A). Surprisingly, the intestines (9 × 10^9^ copies/g) and spleen (4 × 10^9^ copies/g) bore a higher viral load than the liver (3 × 10^6^ copies/g) or kidneys (3 × 10^4^ copies/g). This result strongly suggested that the fetus was infected with HEV before delivery. To confirm further the vertical transmission of HEV from mother to fetus, we detected HEV antigens in the liver, kidneys, spleen and intestines via IHC (Fig. 2B). Notably, positive HEV antigen signals were observed in the liver, kidneys, spleen, and intestines of the premature fetus (Fig. 2C) although these organ were yet fully developed (Fig. 2D). Considering that the fetus was immediately parted from his mother after delivery, fecal–oral routes and contact HEV transmission were excluded. The detection of HEV RNA and antigens in the replication sites of the newly born fetus strongly confirmed that HEV was vertically transmitted from the mother to the fetus.

Hematological parameters of the neonate were also determined from 8 to 20 weeks after birth. Unlike in the HEV-infected pregnant rhesus macaques, WBC, RBC, PLT, PCT, LYMPH, and LYMPH% in the neonate were higher than the reference values, but the other parameters were normal (Supplementary Fig. 3). Almost all of these liver enzymes were within normal range, except that elevated ALP and GGT levels were observed in two neonates, which were born from HEV-infected mothers (Supplementary Fig. 4).

### HEV infection in neonates

**Figure 3:**
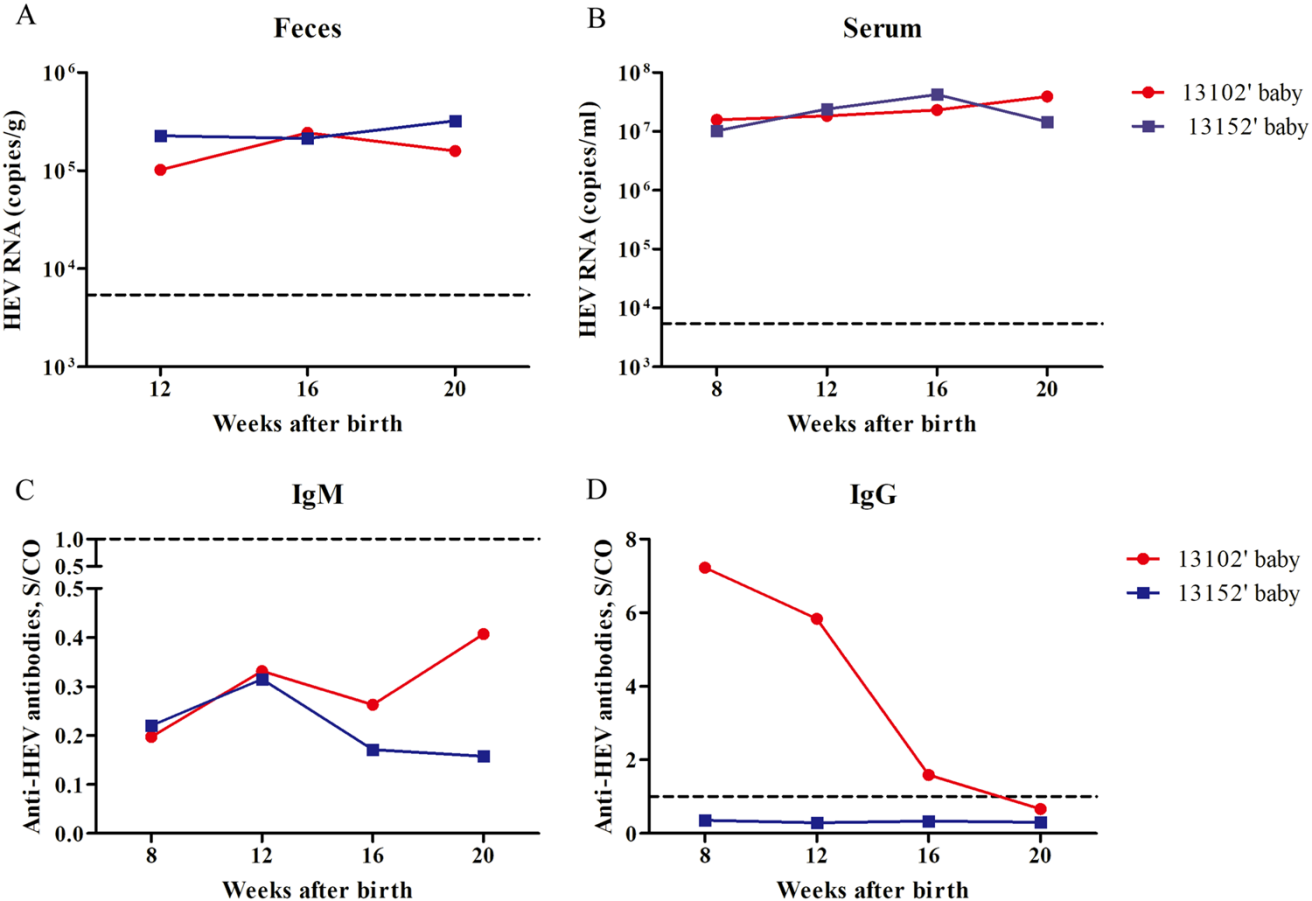
HEV infection in neonates born from HEV-infected mothers. Copy number of HEV in the feces (**A**) or serum (**B**) of neonates born from HEV-infected mothers quantified by qRT-PCR. Anti-HEV IgM (**C**) and IgG (**D**) antibodies determined by ELISA.

More than 45% of neonates born from HEV-infected mothers are positive for HEV^6^. To evaluate the risk of HEV infection in neonates, we collected fecal and blood samples from the newborn rhesus macaques 8 or 12 weeks to 20 weeks after birth. HEV RNA was detected in the feces of two neonates (Fig. 3A). Moreover, viremia was observed in these two newborn macaques whose mothers were infected with gt4 HEV during the third trimester of pregnancy (Fig. 3B). Shedding of HEV viruses in the fecal samples and ongoing viremia strongly indicated active HEV infection in neonates.

The answer to the question of how long maternally transferred antibody lasts and neutralized in neonates remains elusive. In the present study, HEV-specific IgG and IgM antibodies were determined in the two neonates. Although HEV RNA was positive in the blood and fecal samples, the HEV-specific IgM antibody was always negative (Fig. 3C). Only one of the two newborns obtained the maternally transferred antibody, but the level of this antibody sharply declined 8 weeks later and became completely negative at 20 weeks (Fig. 3D). Similarly, the maternally transferred antibody in the newborns of gt1 HEV (SAR-55)-infected rhesus macaques disappeared within 15 weeks after birth^24^. Therefore, the protection conferred by maternally transferred antibody was ineffective and could not neutralize HEV.

### Adverse pregnancy outcomes caused by HEV infection

**Figure 4:**
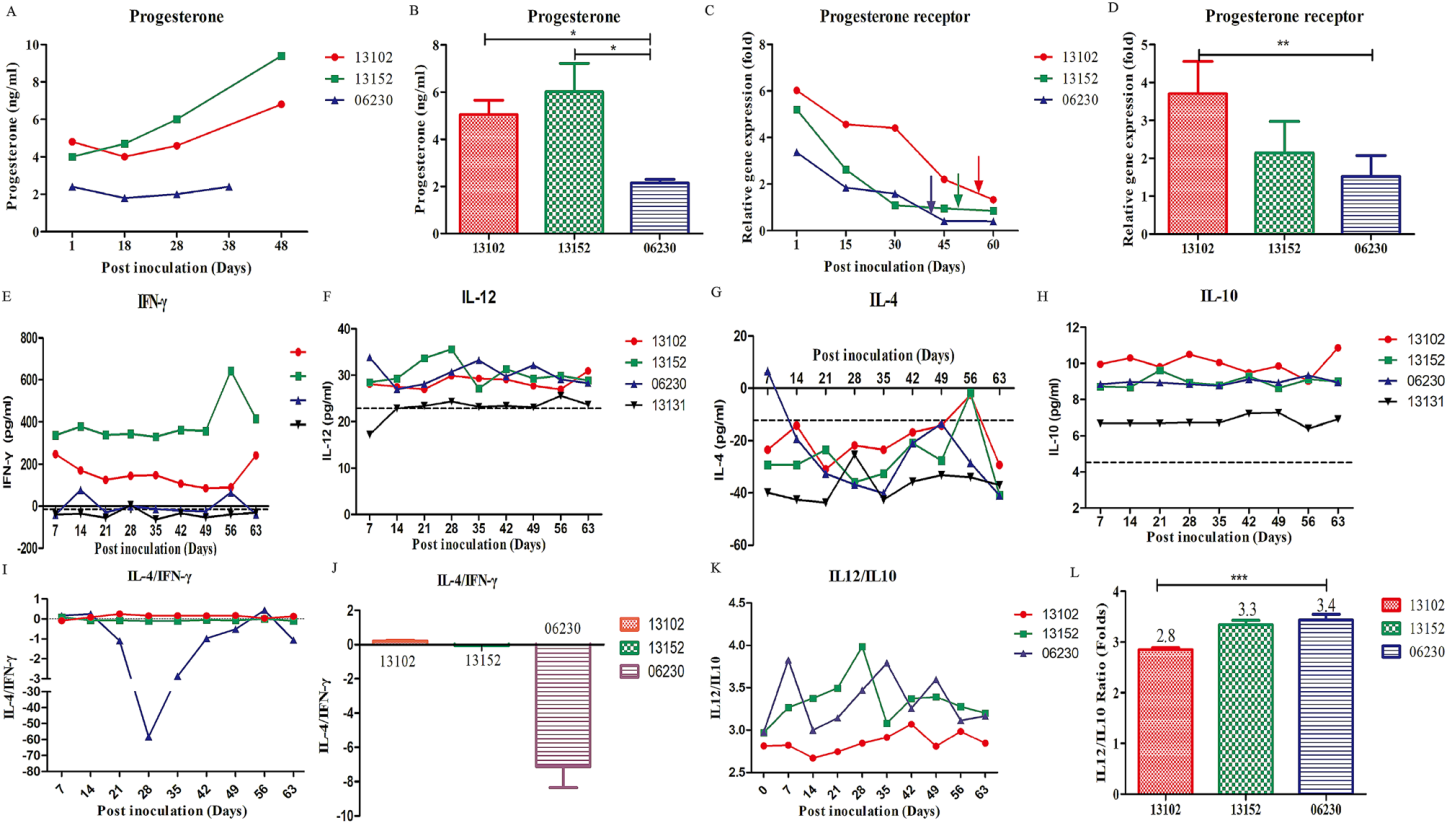
Adverse pregnancy outcomes caused by HEV infection. The concentration of progesterone in HEV-infected pregnant rhesus macaques at indicated times points (**A**). The concentration (mean value) of progesterone during the whole pregnancy (**B**). The relative expressions of progesterone receptor in HEV-infected pregnant rhesus macaques were normalized with non-pregnant rhesus macaque at indicated time points (**C**). The folds of progesterone receptor (mean value) during the whole pregnancy (**D**). The levels of IFN-γ, IL12, IL4 or IL10 were determined in these HEV infected pregnant or non-pregnant rhesus macaques (**E**–**H**). The ratio of IL4/IFN-γ using the concentration of IL4 or IFN-γ in each indicated time points (**I**). The ratio of IL4/IFN-γ using the mean value of IL4 or IFN-γ during the whole pregnancy (**J**). The ratio of IL12/IL10 using the concentration of IL12 or IL10 in each indicated time points (**K**). The ratio of IL12/IL10 using the mean value of IL12 or IL10 during the whole pregnancy (**L**). Arrow indicated the time of delivery.

The female steroid hormones estrogen and progesterone play essential roles in maintaining a healthy pregnancy and ensuring a safe delivery at term^25^. Decline in progesterone levels may be responsible for preterm labor. In the present study, the total level of progesterone in the serum samples during pregnancy was significantly lower in 06230#, which gave birth to a preterm baby, than that in the two other HEV-infected rhesus macaques with normal delivery (Fig. 4A,B). Progesterone level decreased in HEV-infected 06230# macaques from 18 dpi to delivery (Fig. 4A). By contrast, HEV-infected 13152# and 13102# macaques with normally increasing progesterone levels underwent normal delivery. The decrease in progesterone levels might have contributed to preterm birth in HEV-infected rhesus macaques.

Progesterone binds to its receptor, progesterone receptor, to maintain the entire pregnancy. A decrease in the levels of progesterone receptor causes adverse pregnancy outcomes^5^. Administration of progesterone receptor antagonists, such as antiprogestine mifepristone (RU-486), induces cervical ripening, spontaneous abortion, and labor in animals and humans^26^. Remarkably, the expression of progesterone receptor was significantly lower in preterm birth 06230# macaques than in the two other HEV-infected rhesus macaques with normal delivery (Fig. 4C,D).

Preterm birth is a syndrome attributable to multiple pathological processes, such as infection, vascular disorders, decidual senescence, uterine overdistension, and decline in progesterone action^27^. In addition, imbalance of Th1/Th2 cytokines plays a vital role in spontaneous abortion^28^. Normal pregnancy is associated with a Th2-biased cytokine profile, whereas Th1-biased immune status is prone to abortion^28^. Thus, the levels of Th1 cytokines (IL12 and IFN-γ) and Th2 cytokines (IL4 and IL10) were determined to evaluate the shift in Th1/Th2 cytokines in HEV infected pregnant rhesus macaques (Fig. 4E–H). Interestingly, a remarkable Th1-biased immune status was observed in HEV infected rhesus macaque with preterm birth compared with the two other HEV infected rhesus macaques with normal delivery (Fig. 4I–L). Therefore, the low progesterone concentration, decline in progesterone receptor expression, and abortion-prone Th1-biased immune status resulted in poor pregnancy outcomes.

### Suppressed innate immune response during pregnancy promoted HEV replication

The innate immune responses of hosts are activated once HEV infection occurs^29,30^. However, the elaborate immune responses in vivo, especially during pregnancy, are largely unknown. To describe clearly the changes in host defenses induced by HEV infection during pregnancy, we determined the expression of ISGs in blood samples after HEV infection. The entire infection process was divided into three stages: acute infection during pregnancy (before 7 dpi), tolerance before delivery (8 dpi to delivery), and clearance after delivery (after delivery). The expressions of ISGs in the blood samples of each rhesus macaque collected before HEV infection were quantified and served as baseline.

**Figure 5:**
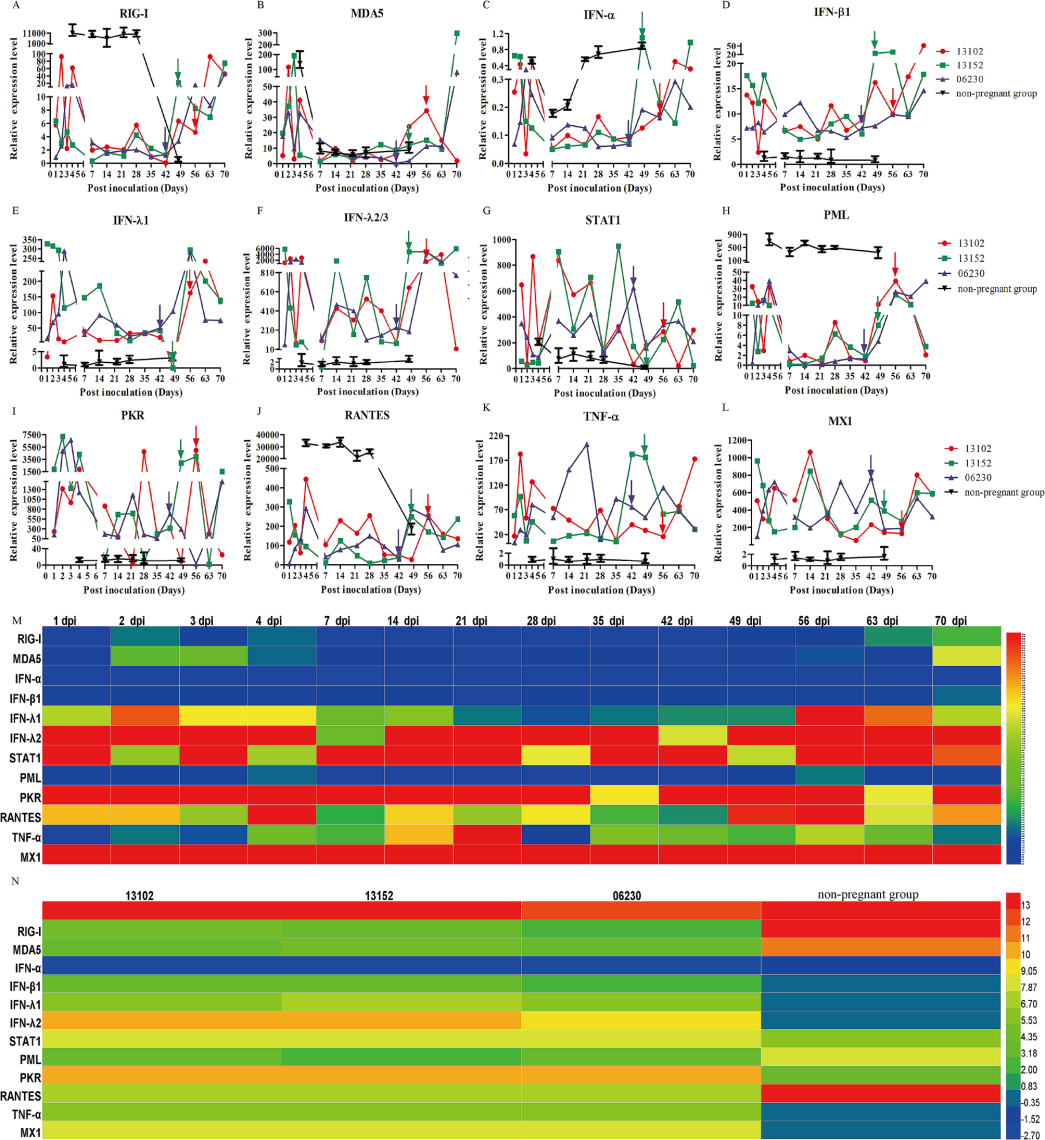
ISGs expressions in HEV-infected pregnant or non-pregnant rhesus macaques. The gene expression of ISGs in the plasma of HEV-infected pregnant or non-pregnant rhesus macaques. The gene expression of 12 ISGs were quantitated by qRT-PCR and normalized with pre-infection (**A**–**L**). The day of farrowing was highlighted by arrow. The relative expression of ISGs in pregnant rhesus macaques was compared with non-pregnant rhesus macaques. Heatmap was generated for the different parameters analyzed at indicated time points (**M**) or individuals (**N**).

RIG-I, the most important PRR during virus infection, was determined after HEV infection. HEV infection induced a substantial increase in RIG-I gene expression during acute infection at the early stage from 1 to 7 dpi with an average of 17.62 ± 12.29-fold increase compared with uninfected rhesus macaques (Fig. 5A). During pregnancy, the maternal immune system is modified to accommodate the fetus. Thus, the expression of RIG-I was maintained at a low level (3.82 ± 4.13-fold higher than pre-infection, p = 0.1144) from 8 dpi to delivery. After delivery, the expression of RIG-I sharply increased (26.89 ± 20.59-fold higher than pre-infection, p = 0.3398) (Fig. 5A). By contrast, the expression of RIG-I in non-pregnant rhesus macaques persistently increased until HEV completely cleared at 49 dpi (7460.70 ± 3421.28-fold higher than pregnant rhesus macaques, p = 0.0007, Fig. 5A). Results indicated that RIG-I expressions were significantly activated during HEV infection but suppressed during pregnancy.

RIG-I and MDA5 are the main members of RLRs that recognize viral RNA. Similarly, MDA5 expression was highly activated in HEV-infected non-pregnant rhesus macaques than pregnant rhesus macaques (25.72 ± 32.61-fold, p = 0.0056) compared with uninfected rhesus macaques (Fig. 5B). Like RIG-I expression, MDA5 expression was activated at the acute infection stage from 1 to 7 dpi (42.69 ± 22.97-fold higher than pre-infection, p = 0.8644), inhibited during pregnancy (8 dpi to delivery, 10.85 ± 9.81-fold, p = 0.4955), and increased by 38.50 ± 51.40-fold (p = 0.4308) once the baby was born (Fig. 5B).

RIG-I and/or MDA5 interact with mitochondrial antiviral signaling to initiate the transcription and production of IFNs, such as type I IFNs, IFN-α, and IFN-β. The IFN-α expression was elevated in HEV-infected pregnant and non-pregnant rhesus macaques (Fig. 5C). IFN-α exhibits efficient anti-HEV activity both in vitro and in vivo^31^. Thus, the higher IFN-α expression might have contributed to the lower viral titer in non-pregnant rhesus macaques than in pregnant rhesus macaques. Surprisingly, of IFN-β expression was evidently inhibited in HEV-infected non-pregnant rhesus macaques. This result was consistent with that of in vitro experiments^32,33^. However, a remarkable increase in IFN-β expression was induced in pregnant rhesus macaques than in non-pregnant group (11.65 ± 5.82 versus 1.14 ± 0.23, p < 0.0001). Similarly, IFN-β expression was moderately inhibited in pregnant rhesus macaques after acute infection until delivery (Fig. 5D).

Persistent HEV infection does not activate IFN-I antiviral responses but upregulates IFN-III responses^34^. In the present study, HEV RNA was persistently positive for more than 6 months in HEV-infected pregnant rhesus macaques, which was longer than the definition of chronic infection of 3 months (Fig. 1). The responses of type III IFN (IFN-λ1 and IFN-λ2/3) notably increased in HEV-infected pregnant rhesus macaques by 106 ± 71.8-fold (p = 0.027) for IFN-λ1 and 1234.79 ± 955.17-fold (p < 0.0001) for IFN-λ2/3 (Fig. 5E,F). Meanwhile, almost no response was observed in HEV-infected non-pregnant rhesus macaques. Like the other ISGs, IFN-λ1 and IFN-λ2/3 were activated during HEV infection and suppression before delivery but were immediately restored after delivery.

RIG-I activates two distinct ISG categories, namely, JAK-STAT-dependent and JAK-STAT-independent, which contribute to anti-HEV activity^30^. STAT1 expression was also determined in this study. Interestingly, STAT1 expression was evidently and highly activated during pregnancy than non-pregnant group (301.11 ± 175.67-fold versus 89.86 ± 55.14-fold, p = 0.0158) (Fig. 5G). STAT1 expression was also activated in Gt4 HEV-infected gorillas^29^, indicating that the JAK/STAT signal pathway was activated and played a critical role in conferring protection against HEV infection. Surprisingly, STAT1 expression was not frustrated before delivery but slightly decreased after delivery when viral titer declined.

The promyelocytic leukemia (PML) protein, which is an IFN-I-induced gene product and a member of the tripartite motif family, modulates the transcriptional activity of viruses^35^. PML can inhibit HIV and HSV replication as part of the IFN-I-mediated response^35,36^. However, PML response to HEV infection is rarely reported. In the present study, PML was evidently activated in HEV-infected rhesus macaques, and the activation was higher in HEV-infected non-pregnant rhesus macaques than in pregnant rhesus macaques (482.52 ± 119.25-fold versus 11.42 ± 10.73-fold, p = 0.0002; Fig. 5H). PML activation was lower before delivery compared with during acute infection and after delivery .

The dsRNA-activated protein kinase R (PKR) plays a major role in vertebrate host immunity against viral infection. PKR is activated by viral double-stranded RNA and subsequently phosphorylates the translation factor eukaryotic initiation factor 2α^37^. Interestingly, PKR was highly activated during HEV infection during pregnancy but was barely detectable in non-pregnant rhesus macaques (1377.43 ± 1293.52-fold versus 12.32 ± 1.64-fold, p = 0.0023, Fig. 5I). PKR expression in pregnant rhesus macaques increased until the end of the experiment (Fig. 5).

HEV infection stimulates a high level of RANTES but a low level of TNF-α in vitro^39^. In HEV-infected rhesus macaques, RANTES expression was significantly activated in non-pregnant rhesus macaques (23,890.13 ± 10,611.52-fold) but was only 149.63 ± 73.46-fold in pregnant rhesus macaques (p = 0.0042, Fig. 5J). However, TNF-α expression was inhibited in HEV-infected non-pregnant rhesus macaques but significantly activated in HEV-infected pregnant rhesus macaques (63.44 ± 28.93-fold versus 0.80 ± 0.15-fold, p = 0.0008, Fig. 5K).

The expressions of the antiviral genes IFITM1 and MX1 are correlated with STAT1 expression in B cells and monocytes. Although HEV infection induces the expression of MX1 in vitro and in vivo^38^, no obvious effect on HEV-infected non-pregnant rhesus macaques was observed (Fig. 5L). By contrast, MX1 expression in HEV-infected pregnant rhesus macaques was significantly activated (391.21 ± 160.76-fold, p < 0.0001). MX1 expression slightly decreased in HEV-infected rhesus macaques before delivery. Finally, the difference of ISGs expression during HEV infection was shown in Fig. 5M. Furthermore, immune tolerance with lower expression of ISGs was observed in these pregnant rhesus macaques compared with non-pregnant rhesus macaques (Fig. 5N).

## Discussion

HEV infection has emerged as a serious global public health issue, especially for pregnant women^2^. During pregnancy, the immune system is altered to protect against the mother’s immune rejection. However, the tolerant immune system is prone to pathogenic infection. Lower lymphocyte proliferation response to phytohemagglutinin (PHA) was observed in pregnant women with acute hepatitis E compared with non-pregnant patients with HEV or healthy pregnant women^40^. HEV infection causes adverse pregnancy outcomes, including fulminant hepatic failure, membrane rupture, spontaneous abortion, and stillbirth^23,41^. Althousgh genotype 4 HEV infection is more severe than genotype 3 HEV infection^42^, its infection in pregnant women is usually ignored.

The pathogenesis of HEV infection in pregnant women remains uncertain mainly because of the lack of a suitable animal model. HEV genotype 1 in pregnant rhesus macaques and HEV-3 in gilts have failed to provide clear evidence of vertical transmission or adverse pregnancy outcomes^43,44^. Rhesus macaque is one of the best animal models for HEV study. Fortunately, genotype 4 HEV infected pregnant rhesus macaque models were successfully established in the present study. The successful establishment provided a valuable chance to explore the underlying mechanism of HEV infection.

In this study, higher viral titers were detected in the fecal and blood samples of pregnant rhesus macaques than in non-pregnant rhesus macaque; this result was consistent with the classical clinical symptoms of HEV infection in pregnant women^45^. In our previous study, we confirmed estrogen-promoting HEV replication in cells when estrogen analogs are supplied (17β-estradiol or diethylstilbestrol)^45^. Estradiol level is positively correlated with viral titer. However, the levels of progesterone and progesterone receptor decreased in HEV-infected pregnant rhesus macaques with premature delivery and fetal death. Substantial deregulation of progesterone receptor was also found in HEV-related FHF pregnant women^5^. The high estrogen and low progesterone levels caused by HEV infection during pregnancy might have contributed to the adverse pregnancy outcomes.

HEV infection alters the expression of ISGs in vitro and in vivo^30^. IFNs are broad antiviral cytokines by transcription multiple ISGs. RIG-I is the most important anti-HEV ISG against HEV regardless of INF production. RIG-I and MDA5 over-expression effectively inhibits HEV viral RNA levels in Huh7.5.1 cells^30^. RIG-I expression was remarkably stimulated in HEV-infected non-pregnant rhesus macaques but frustrated in HEV-infected pregnant rhesus macaques, especially at the tolerance stage after acute infection and before delivery. Frustrated RIG-I expression rarely produces IFN-I (IFN-α and IFN-β). Similarly, diminished, unaltered antiviral responses, or dramatically reduced cytokines were reported in pregnant women with acute hepatitis E^46^. Consequently, the viruses in pregnant rhesus macaques could not be efficiently and immediately eliminated, thus prolonging HEV duration. Down regulated innate immune response during pregnancy plays an important role in persistent HEV infection. HEV-persistent infection is associated with IFN-III^34^. IFN-III was considerably activated in HEV-infected pregnant rhesus macaques but was barely detected in acute HEV-infected non-pregnant rhesus macaques. However, the elevated IFN-III activation was insufficient to eliminate HEV and thus the virus persisted for 6 months.

Vertical HEV transmission was first reported in 1995^47^, but its mechanism remains unclear. Kumar et al. reported high risks of mother-to-infant vertical HEV transmission in 469 pregnant women with 100% mother-to-infant transmission^48^. In the present study, HEV RNA and HEV antigens were detected in the liver, spleen, kidneys, and intestines of the dead fetus of an HEV-infected mother. This result directly confirmed vertical HEV transmission. The tissues were immediately collected after delivery to avoid contact transmission from the mother. Unfortunately, cord blood and neonatal samples were not obtained because of the mother’s protection.

Fecal–oral transmission is the main route of HEV transmission. HEV can be transmitted from milk and cause acute infection^20^. Thus, fecal–oral or contact transmission could not be excluded because of positive HEV RNA signal in the serum and stool samples from 8 or 12 weeks to 20 weeks. However, acute HEV infection in newborn macaques did not evoke adaptive immunity as they were negative to IgM antibody. Anti-HEV IgG antibody decreased in one of the two newborns and completely disappeared at 20 weeks; hence, the antibody might have been acquired from the mother. Although the newborn was positive to anti-HEV IgG antibody with a high titer (S/CO = 7.22), HEV RNA was detected in the fecal and blood samples, which indicated that the maternally transferred antibody was insufficient to confer protection against HEV infection.

Cytokines play important roles in regulating innate, humoral, and cellular immune responses against viral infection. A clear shift is observed in Th1:Th2 cell paradigm during pregnancy, with a definite skew toward Th2 cells. Normal human pregnancy is associated with a Th2-biased cytokine profile^5^. HEV-related FHF pregnant women with high fetal and maternal mortality were altered maximally toward the Th1-biased immune state^5^. Furthermore, the Th1-biased cytokine profile was observed in HEV-infected pregnant BALB/c mice^22^. In the present study, a Th1-biased immune state was found in HEV-infected pregnant rhesus macaques, and this state probably contributed to premature delivery. The remarkable alternation of Th1 and Th2 balance may partly explain the poor pregnancy outcomes.

## Methods

### Ethics statement

The animal experiment was approved by the Animal Care and Use Committee of Institute of Medical Biology, Chinese Academy of Medical Sciences and Peking Union Medical College. All procedures were performed under ketamine anesthesia by trained personnel under the supervision of veterinary staff. All efforts were made to ameliorate the welfare of the animals and minimize their suffering in accordance with the recommendations cited in “Weatherall report for the use of non-human primates”. Rhesus macaques were housed individually and fed with complete formula food. Details of the rearing environment are described in our previous study^49^. All rhesus macaques included in the present study were healthy and negative for HEV RNA or anti-HEV immunoglobulin G (IgG) and immunoglobulin M (IgM) antibodies.

### HEV infection protocol

Three pregnant rhesus macaques (13102#, 13152#, and 06230#) in their third trimester of pregnancy were intravenously injected with gt4 HEV (KM01 strain, derived from HEV-infected swine stool, GenBank: KJ155502, 5.1 × 10^5^ copies/mL). Three non-pregnant rhesus macaques (13131#, 13279# and 13329#) were intravenously injected with the same gt4 HEV strain and served as non-pregnant controls. The mean ± SD of all these three non-pregnant controls were present and named as non-pregnant group or 13131#. Stool samples were collected twice weekly, whereas blood samples were collected once every week. Tissue samples from dead fetus were immediately collected and stored in liquid nitrogen for RNA detection or fixed with 10% neutral buffered formalin for histopathology analysis.

### Clinical parameters

Blood was collected from HEV-infected rhesus macaques by using EDTA-K2 tubes. Hematological parameters were analyzed using an automatic hematological analyzer (XT2000i SYSMEX, Kobe, Japan): number of white blood cells (WBC), number of red blood cell (RBC), hemoglobin (HGB), hematocrit (HCT), platelets (PLT), mean platelet volume (MPV), plateletcrit (PCT), mean corpuscular volume (MCV), mean corpuscular hemoglobin (MCH), mean corpuscular hemoglobin concentration (MCHC), neutrophil percentage (NEUT%), monocyte percentage (MONO%), eosinophil percentage (EO%), basophilic leukocyte percentage (BASO%), lymphocyte percentage (LYMPH%), neutrophil (NEUT#), lymphocyte (LYMPH#), monocyte (MONO#), eosinophil (EO#), basophil (BASO%#), red blood cell volume distribution width-SD (RDW-SD), red blood cell volume distribution width-CV (RDW-CV), plate volume distribution width (PDW), and platelet large cell ratio (P-LCR).

Meanwhile, serum was separated and used to measure chemical parameters by using a serum chemistry analyzer (BS-200 Mindray, Shenzhen, China): alanine aminotransferase (ALT), aspartate aminotransferase (AST), alkaline phosphatase (ALP), total protein (TP), albumin (ALB), globulin (GLB), total bilirubin (T-BIL), direct bilirubin (D-BIL), indirect bilirubin (I-BIL).

### Gene quantification

Total RNA was extracted from the supernatant of feces, serum, blood, or tissues by using TRIzol reagent (Invitrogen, Carlsbad, USA) according to the manufacturer’s instructions. RNA was reverse-transcribed to cDNA by using Avian Myeloblastosis Virus (AMV) reverse transcriptase with HEV-specific or random hexamer primers. HEV titer was quantitated using SYBR Green-based quantitative real-time polymerase chain reaction (qRT-PCR) with HEV-specific primers according previous studies^20,50^.

The expression of ISGs, including RIG-1, MDA5, PKR, RANTES, IFN-α, IFN-β1, IFN-λ1, IFN-λ2, STAT1, TNF-α, MX1, and PML, was quantified from peripheral blood by using specific primers described in previous studies^30,39^ and listed in Table 1. GAPDH was used as the housekeeping control. Relative gene expression was calculated by the 2^−(△Ct of gene − △Ct of GAPDH)^ method, where *Ct* is the threshold cycle.

**Table 1:** The primers used in this study.

Primers	Sequences (5′ to 3′)	References
RIG-I	CACCTCAGTTGCTGATGAAGGC	^30^
	GTCAGAAGGAAGCACTTGCTACC	
IFIH1(MDA5)	GCTGAAGTAGGAGTCAAAGCCC	^30^
	CCACTGTGGTAGCGATAAGCAG	
IFN-α	TGGGCTGTGATCTGCCTCAAAC	^30^
	CAGCCTTTTGGAACTGGTTGCC	
IFN-β1	CTTGGATTCCTACAAAGAAGCAGC	^30^
	TCCTCCTTCTGGAACTGCTGCA	
IFN-λ1	GGAAGACAGGAGAGCTGCAACT	^30^
	AACTGGGAAGGGCTGCCACATT	
IFN-λ2	TCGCTTCTGCTGAAGGACTGCA	^30^
	CCTCCAGAACCTTCAGCGTCAG	
STAT1	ATGGCAGTCTGGCGGCTGAATT	^30^
	CCAAACCAGGCTGGCACAATTG	
PML	CCGTCATAGGAAGTGAGGTCTTC	^30^
	GTTTTCGGCATCTGAGTCTTCCG	
PKR	GAAGTGGACCTCTACGCTTTGG	^30^
	TGATGCCATCCCGTAGGTCTGT	
MX1	GGCTGTTTACCAGACTCCGACA	^30^
	CACAAAGCCTGGCAGCTCTCTA	
RANTES	CCTGCTGCTTTGCCTACATTGC	^30^
	ACACACTTGGCGGTTCTTTCGG	
TNF-α	CAGAGGGAAGAGTTCCCCAGGGACC	^39^
	CCTTGGTCTGGTAGGAGACGG	
GAPDH	TGTCCCCACCCCCAATGTATC	^30^
	CTCCGATGCCTGCTTCACTACCTT	

### Anti-HEV antibodies and cytokine determination

Anti-HEV IgG and IgM antibodies were tested via enzyme-linked immunosorbent assay (ELISA) (WanTai, Beijing China) according to the manufacturer’s protocol. Levels of Th1 cytokines (interleukin 12 [IL12] and interferon gamma [IFN-γ]) and Th2 cytokines (IL 4 and IL10) in the serum were determined using ELISA kits (4A Biotech, Beijing, China) according to the manufacturer’s instructions.

### Histopathology and immunohistochemistry analyses

Tissues were fixed in 10% neutral buffered formalin and embedded in paraffin. Specimens were cut into 3 µm serial sections. Standard hematoxylin and eosin staining was performed, and the specimens were examined under a microscope.

Immunohistochemistry (IHC) was performed according to our previous studies^22^. In brief, tissues were deparaffinized, hydrated, water bath-heated for antigen retrieval, and then blocked with the addition of 3% hydrogen peroxide for 15 min. Tissue sections were incubated for 2 h at 37 °C with primary antibody (HEV ORF2, 1:250 dilution, MAB8003, Merck Millipore, German), washed with PBS, and then incubated with HRP-labelled Goat anti-mouse IgG (H + L) antibody. The paraffin sections were inoculated with PBS instead of HEV-specific antibody and served as the negative control. The reaction was visualized using DAB (Abcam, Cambridge, UK) as chromogen. The slides were sealed with neutral balsam, inspected, and photographed under a microscope (Nikon, E200, Japan).

### Statistical analysis

All experiments were performed three times. GraphPad Prism 5.01 software was used for statistical analysis. Student *t*-test was used to determine the significance of differences between two groups. A *P* value of < 0.05 was considered statistically significant.

## Supplementary information

Supplementary Information.
