# Supplementary material for: Vertical transmission of hepatitis E virus in pregnant rhesus macaques
Source: Sci Rep. 2020 Oct 15;10:17517. doi: 10.1038/s41598-020-74461-7 (PMC7567892; doi:10.1038/s41598-020-74461-7)

Supplementary file to:

Vertical transmission of Hepatitis E virus in pregnant rhesus macaques

Wenhai Yu[1#], Xianhui Hao[2#], Yi Li[2#], Chenchen Yang[2], Yunlong Li[2], Fen Huang[2]*, Zhanlong He[1]*

[1]Institute of Medical Biology, Chinese Academy of Medical Sciences and Peking Union Medical College, Kunming, PR China;

[2]Medical Faculty, Kunming University of Science and Technology, Kunming, PR China.

Supplementary Fig.1

The levels/activities of liver enzymes of HEV infected pregnant or non-pregnant rhesus macaques.

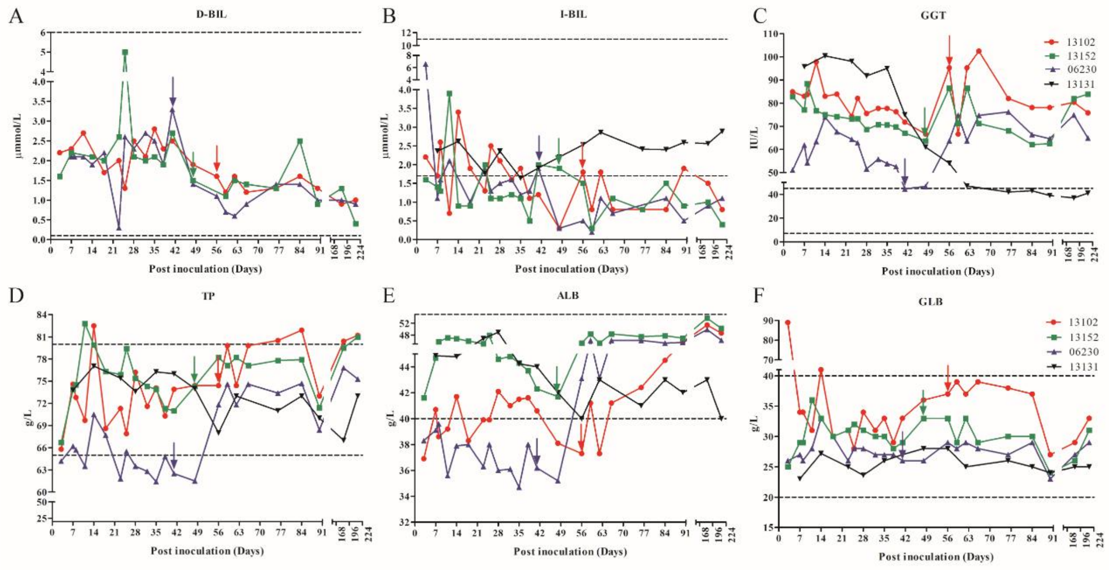

Supplementary Fig.2

Hematological parameters of HEV infected pregnant or non-pregnant rhesus macaques.

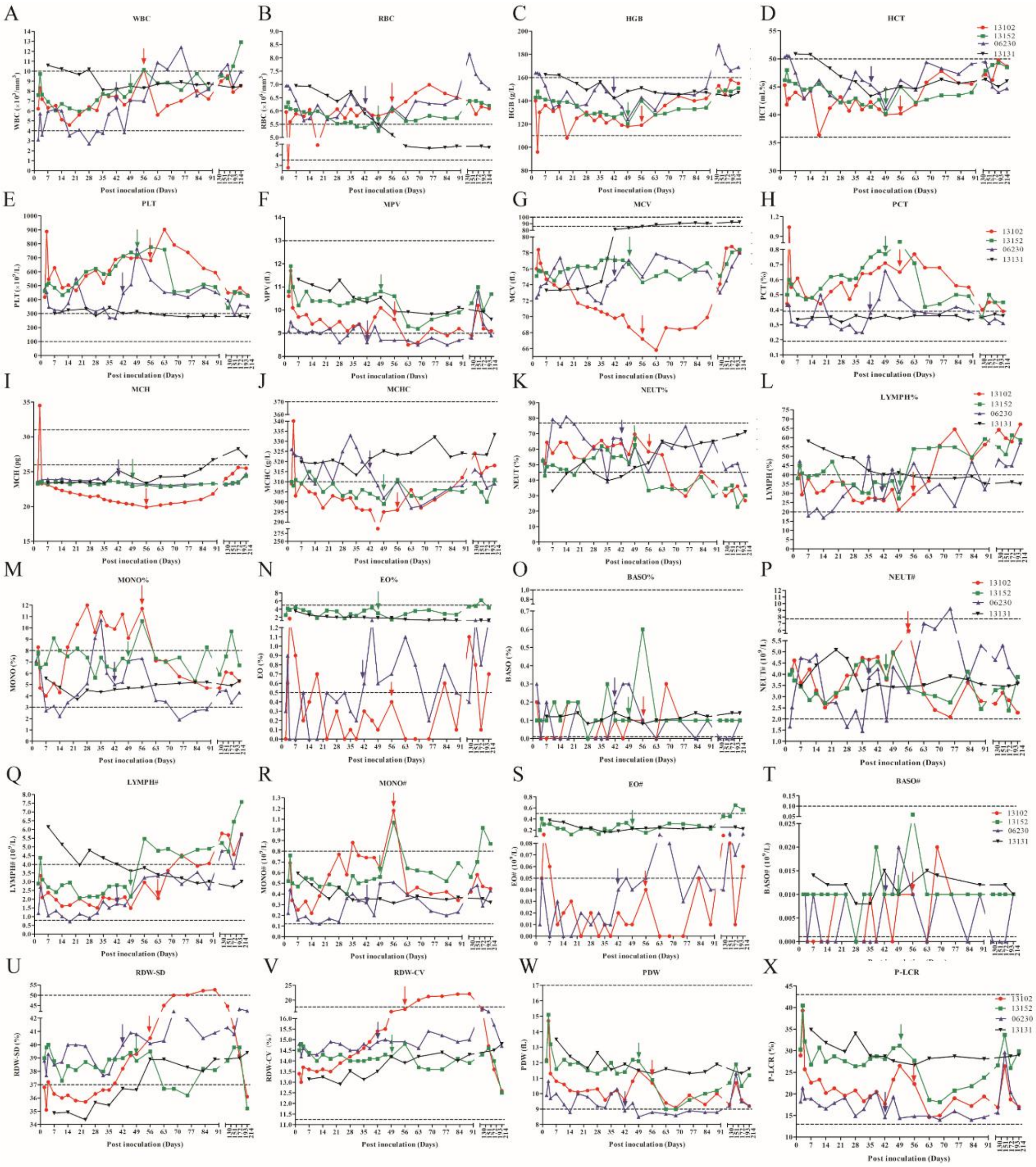

Supplementary Fig.3

Hematological parameters of neonates born from HEV infected mothers.

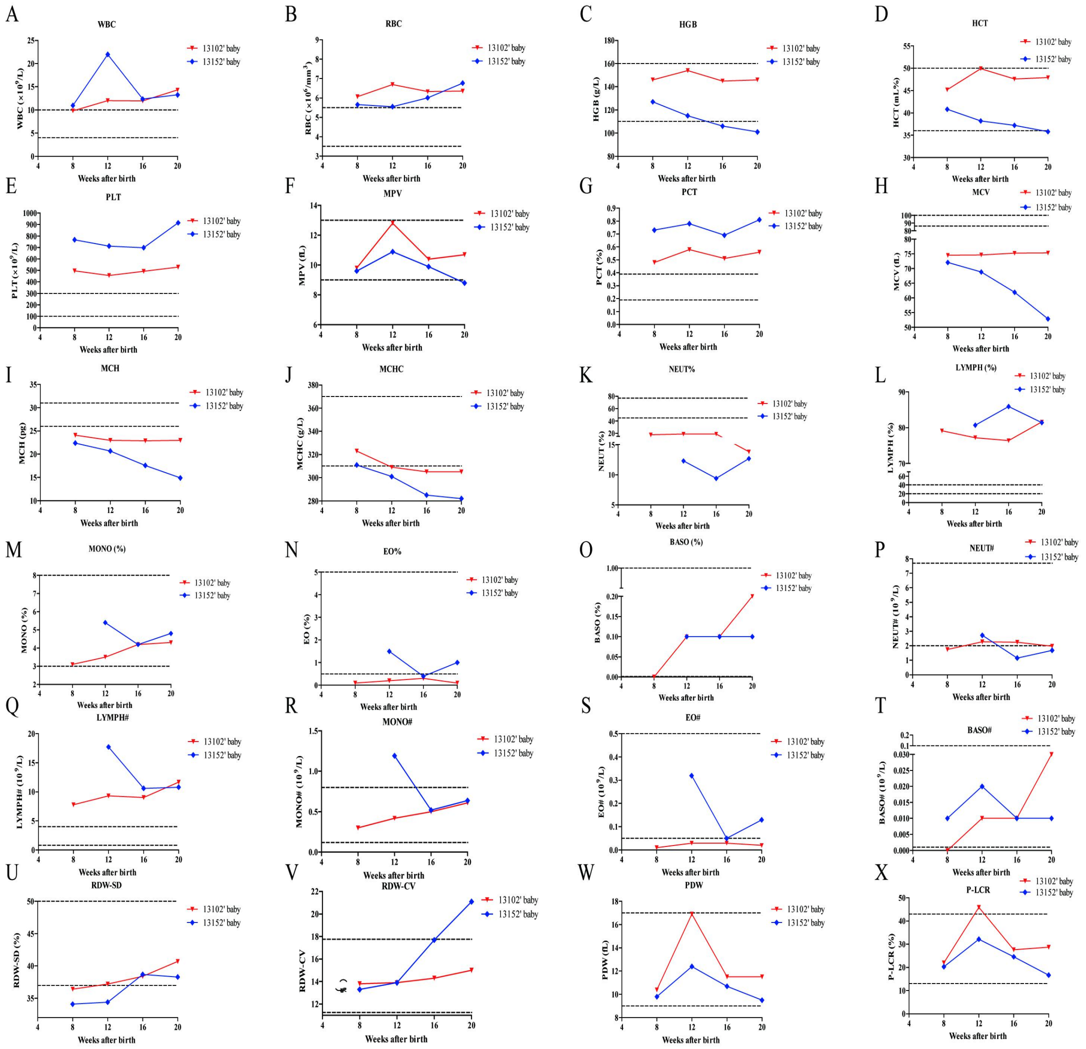

Supplementary Fig.4

The levels/activities of liver enzymes of neonates born from HEV infected mothers.

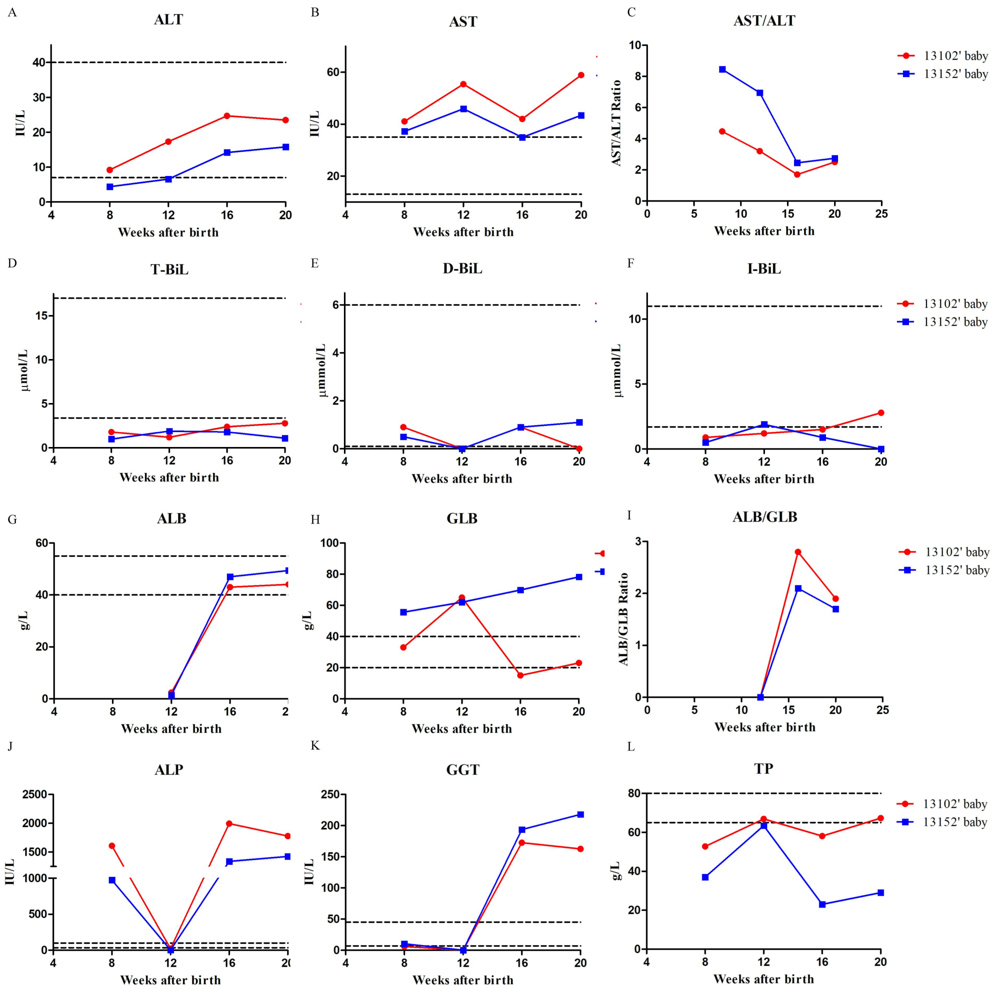

Supplement: Supplementary file 1 — Supplementary Information. [file 41598_2020_74461_MOESM1_ESM.pdf]
